# Purtscher-like retinopathy associated with acute pancreatitis

**DOI:** 10.1590/S1516-31802005000600008

**Published:** 2005-11-01

**Authors:** Clayton Rocha Lara Carrera, Leandro Mont'Alverne Pierre, Flavio Mac Cord Medina, Paulo de Tarso Ponte Pierre-Filho

**Keywords:** Pancreatitis, Retina, Visual acuity, Case reports, Vision disorders, Pancreatite, Retina, Acuidade visual, Relatos de casos, Transtornos da visão

## Abstract

**CONTEXT:**

Purtscher-like retinopathy with bilateral loss of vision is a rare and severe complication that may follow acute pancreatitis.

**CASE REPORT:**

The case of a 35-year-old patient with acute alcoholic pancreatitis who developed sudden loss of visual acuity is described. The ophthalmoscopic examination revealed diffuse retinal whitening of the posterior pole with confluent cotton-wool spots. Fluorescein angiogram showed retinal arteriolar occlusion. The findings were compatible with Purtscher-like retinopathy. Computed tomography of the abdomen demonstrated enlarged liver and pancreas with edema and inflammation. The pathogenesis of this form of retinopathy still remains uncertain and there is no specific treatment available.

## RESUMO

**CONTEXTO:**

Retinopatia de Purtscher com redução da visão bilateral é uma rara e grave complicação da pancreatite aguda.

**RELATO DE CASO:**

O caso de um paciente de 35 anos com pancreatite alcoólica aguda que apresentou diminuição súbita da acuidade visual é descrito. Oftalmoscopia revelou pólo posterior esbranquiçado devido à presença de manchas algodonosas confluentes. Angiografia fluoresceínica mostrou oclusão arteriolar retiniana. Os achados eram compatíveis com retinopatia de Purtscher. Tomografia computadorizada do abdômen demonstrou fígado e pâncreas aumentados, com edema e inflamação. A patogênese desta forma de retinopatia ainda é incerta e não há tratamento específico disponível.

## INTRODUCTION

Purtscher's retinopathy was first described in 1910 in patients suffering severe head injury who presented sudden loss of vision within hours of sustaining their injury. This retinopathy is characterized by generalized retinal and macular edema with associated superficial hemorrhages. Since the original description, other conditions associated with Purtscher's retinopathy have been reported, including acute pancreatitis, lymphoproliferative disorders, chest compression, bone marrow transplantation, fat embolization, Valsalva maneuver and pancreatic adenocarcinoma.^1^

An association between Purtscher's retinopathy and acute pancreatitis was first reported by Inkeles and Walsh in 1975.^2^ The case presented here provides an illustration of a rarely recognized complication of acute pancreatitis that is well documented in the ophthalmological literature but not commonly recognized by physicians of other specialties.

## CASE REPORT

A 35-year-old man with a history of alcohol abuse was referred to the hospital because of epigastric pain that radiated through the back and flanks. On physical examination in the emergency room, he was conscious, alert, adjusted to his surroundings and hemo-dynamically stable. During the initial three hours of hospitalization, he suffered sudden visual impairment.

**Figure 1:**
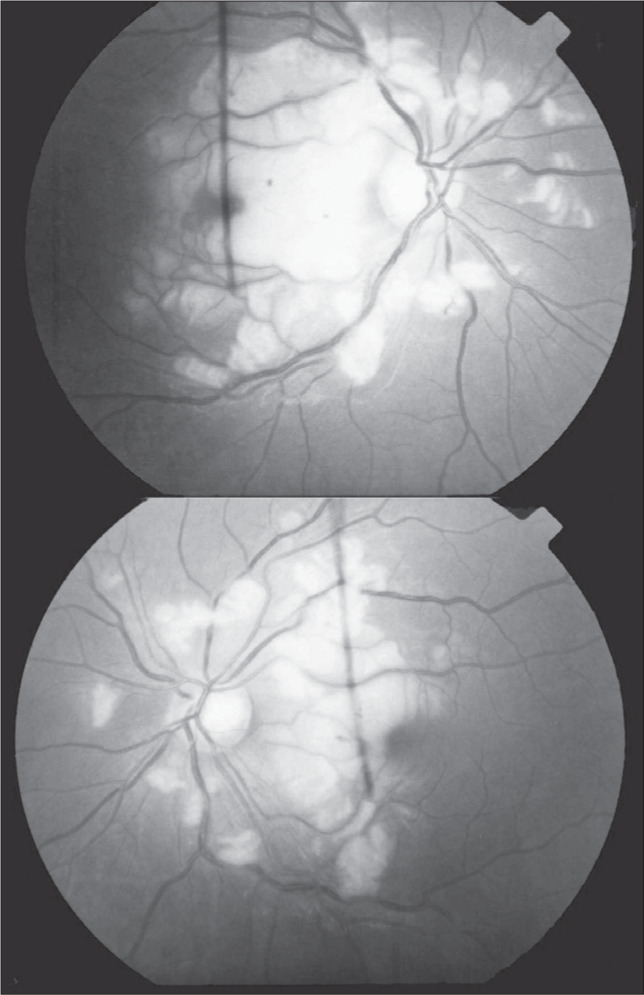
Right (top) and left (bottom) fundus upon first examination showing cotton-wool spots near the blood vessels, retinal whitening and a few superficial hemorrhages, in a 35-year-old man admitted to the emergency room with acute pancreatitis, who suffered loss of visual acuity during examination.

**Figure 2:**
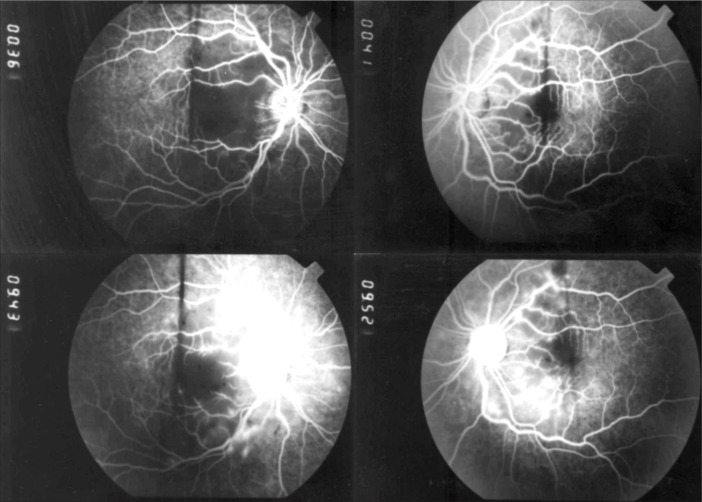
Early (top) and late (bottom) phases of the fluorescein angiogram in a 35-year-old man with acute pancreatitis who suffered loss of visual acuity during clinical examination in the emergency room; note that the late venous phase shows hypofluorescence due to ischemia as well as retinal edema at sites of cotton-wool spots and perivenous staining.

Laboratory blood tests revealed 11 × 10^9^/l polymorphonuclear leukocytes, mean corpuscular volume (MCV) 99 femtoliters (fl), mean corpuscular hemoglobin (MCH) 33.4 picograms (pg), platelets 560,000/ml. Other remarkable values were serum amylase 1810 IU/l, lipase 689 IU/l, alkaline phosphatase 154 IU/l, and γ-glutamyltransferase 277 IU/l. Chest x-ray was normal. Abdominal computed tomography showed an enlarged liver; the pancreas was also enlarged, with edema and inflammation. The patient's best-corrected visual acuity was in counting fingers using both eyes. Slit-lamp examination and intraocular pressures were normal. Ophthalmoscopic examination of the fundus revealed diffuse retinal whitening of the posterior pole with confluent cotton-wool spots. The arterioles were narrowed, and there were a few superficial hemorrhages (Figure 1). Fluorescein angiography showed ischemia due to the retinal artery obstruction and evidence of leakage from vessels in the areas corresponding to the cotton-wool spots seen via fundoscopy (Figure 2).

The findings were compatible with the diagnosis of Purtscher-like retinopathy. The acute pancreatitis was self-limited and subsided spontaneously after five days of conventional treatment. Two months after initial presentation, his vision using the left eye was significantly improved from counting fingers to 20/20 (Snellen chart), whereas the right eye was 20/100. Using fundoscopy, residual retinal edema and cotton-wool spots were visibly more severe in the right eye than in the left.

## DISCUSSION

Purtscher-like retinopathy with bilateral loss of vision is a rare and severe complication that may follow acute pancreatitis. The clinical features of our patient were similar to those of cases of pancreatitis-associated retinopathy previously reported.^2–4^ He also had a long history of alcohol intake. Severity of acute pancreatitis is not associated with the presence of retinopathy. Rapid development of visual disturbance is a common and dramatic presentation of this syndrome, although the severity of visual impairment can vary widely, depending upon the specific areas of retina involved in the pathological changes.^3,4^

The pathogenesis of Purtscher-like retinopathy is a matter for debate. Current evidence suggests that leukocytic emboli form when pancreatic damage releases proteolytic enzymes into the systemic circulation, thereby causing activation of the complement cascade and the formation of C5a-induced leukocyte, platelet, and fibrin aggregates. In clinicopathological studies, occluded retinal arterioles and choroid vessels together with damage to the photoreceptors have been described.^5^ Some of the angiographic and clinical features in the present case suggest that the retina and choriocapillary ischemia occurred due to the occlusion of small arterioles by intravascular microparticles generated by the underlying condition.

It is unclear how frequent Purtscher-like retinopathy is in cases of acute pancreatitis, because the symptoms are often overlooked or misdiagnosed, but fewer than 50 cases have been reported to date. To our knowledge, this is the first case of Purtscher-like retinopathy associated with acute pancreatitis reported in Brazil. This case serves as a reminder that patients with acute pancreatitis can present with systemic manifestations that do not, initially, lead to suspicion of pancreatic disease. It demonstrates a condition that few physicians are familiar with. It remains to be established what the best treatment for these ocular complications would be and the outcome therefore depends upon resolution of the pancreatic disease.

## CONCLUSION

Acute pancreatitis may involve remote organ systems, including the eye. This visual disorder is a rare systemic manifestation of acute pancreatitis that is not correlated with a severe or complicated clinical course.
